# Supplementary material for: The Impact of Repeated Attachment Priming on Paranoia, Mood and Help-Seeking Intentions in an Analogue Sample
Source: Brain Sci. 2021 Sep 22;11(10):1257. doi: 10.3390/brainsci11101257 (PMC8533775; doi:10.3390/brainsci11101257)

Supplementary Material for: *The impact of repeated attachment priming on paranoia, mood, and help-seeking intentions in an analogue sample*

Table S1

Correlations between state variables

Scale	1	2	3	4	5	6	7	8	9	10	11	12	13	14	15	16	17	18	19
**Time 1a**																			
(1) Paranoia	-																		
(2) Positive affect	-.06	-																	
(3) Negative affect	.41**	-.14	-																
(4) Help-seeking	-.19	.25*	-.19	-															
(5) Cognitive fusion	.37**	-.39**	.53**	-.23*	-														
**Time 1b**																			
(6) Paranoia	.83**	-.09	.43**	-.21	.39**	-													
(7) Positive affect	-.00	.85**	-.01	.36**	-.20	-.08	-												
(8) Negative affect	.33**	-.07	.83**	-.15	-.37**	.43**	-.04	-											
(9) Help-seeking	-.16	.25*	.48**	.89**	-.16	-.23*	.41**	-.16	-										
(10) Cognitive fusion	.34**	-.31**	.35**	-.24*	.79**	.44**	-.23*	.50**	-.22*	-									
**Time 3**																			
(11) Paranoia	.72**	-.07	.35**	-.12	.38**	.82**	-.05	.37**	-.17	.43**	-								
(12) Positive affect	-.05	.71**	.83	.17	-.17	-.07	.75**	-.06	.25*	-.19	-.09	-							
(13) Negative affect	.31**	-.04	.67**	-.06	.34**	.42**	-.01**	.72**	-.05	.40**	.50**	-.05	-						
(14) Help-seeking	-.08	.14	-.06	.76**	-.10	-.12	.30**	-.15	.85*	-.23*	-.20	.24*	-.06	-					
(15) Cognitive fusion	.30**	-.23*	.41**	-.03	.57**	.40**	-.16	.36**	-.04	.66**	.53**	-.28**	.66**	-.10	-				
**Time 5**																			
(16) Paranoia	.71**	.08	.26*	-.04	.24*	.76**	.03	.30**	-.14	.31**	.85**	-.04	.44**	-.14	.46**	-			
(17) Positive affect	.06	.65**	.10	.14	-.11	.04	.72**	.03	.23*	-.10	.04	.69**	.09	.17	-.08	-.03	-		
(18) Negative affect	.34**	-.03	.62**	-.10	.36**	.44**	-.06	.70**	-.10	-43**	.39**	.05	.68**	-.07	.41**	.46**	-.01	-	
(19) Help-seeking	-.05	.17	-.05	.72**	.00	-.04	.33**	-.05	.80**	-.07	-.04	.23*	.06	.81**	.06	-.11	.31**	-.05	-
(20) Cognitive fusion	.35**	-.22	.34**	-.04	.56**	.40**	-.19	.33**	-.05	.69**	.53**	-.10	.49**	-.10	.71**	.57**	-.15	.62**	.00

* $p < .05$. ** $p < .01$.

Table S2

Correlations between trait variables

	1	2	3	4	5	6
(1) Paranoia	-					
(2) Attachment anxiety	.28*	-				
(3) Attachment avoidance	.23*	-.18	-			
(4) Depression	.12	.22*	.26*	-		
(5) Anxiety	.17	.42**	.05	.62**	-	
(6) Stress	.27*	.40**	.04	.67**	.68**	-
(7) Cognitive Fusion	.09	.44**	-.02	.47**	.45**	.64**

* p < .05. ** p < .01.

Table S3

Correlations between state difference score (Time 5-Time 1a) variables in mediation

Scale	1	2	3	4
(1) Paranoia	-			
(2) Positive affect	-.35**	-		
(3) Negative affect	.42**	-.27*	-	
(4) Help-seeking	-.35**	.41**	-.28*	-
(5) Cognitive fusion	.49**	-.25*	.56**	-.25*

Note. Time 5 = post-prime day 5; Time 1a = pre-prime day 1.

* $p < .05$. ** $p < .01$.

Exploratory analyses

We explored the impact of repeated priming on trait measures taken on Day 5 to see whether there were any changes in participants' attachment avoidance, attachment anxiety, depression, anxiety, stress, and cognitive fusion between the secure and insecure-avoidant prime groups from Time 1a (pre-prime) to 5 (post final prime) using mixed-model ANOVAs.

Trait attachment anxiety

There was no main effect of prime (F(1, 77)=0.23, p=0.63), a main effect of time (F(1, 77)=7.54, p=0.01, ηp^2=0.09) and a prime by time interaction, (F(1,77)=4.98, p=0.03, ηp^2=0.04) (see Figure S1). The two groups did not differ at baseline (F(1, 77)=1.82, p=0.18) or time 5 (F(1, 77)=0.15, p=0.70). Attachment anxiety decreased from baseline (M=4.69, SD=0.85) to time 5 (M=4.27, SD=1.01) in the secure prime condition (t(41)=3.58, p=0.001, d=0.45), and did not change over time in the insecure-avoidant prime condition (t(36)=0.37, p=0.72).

Trait attachment avoidance

There was no main effect of prime (F(1, 77)=0.01, p=0.94) or time (F(1, 77)=0.08, p=0.78), and no prime by time interaction (F(1,77)=3.07, p=0.08).

Trait depression

There was no main effect of prime (F(1, 77)=0.25, p=0.62) or time (F(1, 77)=1.38, p=0.24), and no prime by time interaction (F(1,77)=0.61, p=0.44).

Trait anxiety

There was no main effect of prime (F(1, 77)=1.27, p=0.26), a main effect of time (F(1, 77)=7.42, p=0.01), and no prime by time interaction (F(1,77)=0.004, p=0.95).

Trait stress

There was no main effect of prime (F(1, 77)=0.79, p=0.38) or time (F(1, 77)=1.29, p=0.26), and no prime by time interaction (F(1,77)=0.09, p=0.76).

Trait cognitive fusion

There was no main effect of prime ($F(1, 77)=1.36$, $p=0.25$), a main effect of time ($F(1, 77)=18.85$, $p<0.001$), and no prime by time interaction ($F(1,77)=0.99$, $p=0.32$).

Figure S1

Change in trait attachment anxiety from baseline (time 1a) to post-prime (time 5)

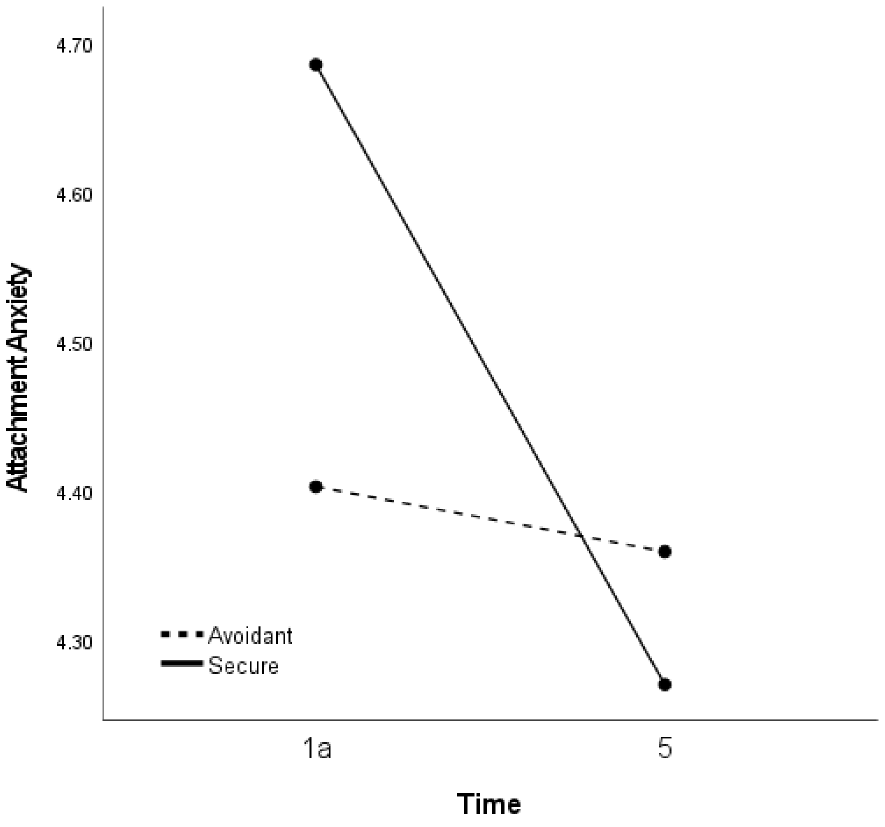

Supplement: Supplementary file 1 [file brainsci-11-01257-s001.zip › brainsci-1332503-supplementary.pdf]
